# Perceptions of stomatherapy graduates regarding in-person and remote education during COVID-19 pandemic

**DOI:** 10.1590/0034-7167-2024-0215

**Published:** 2025-08-08

**Authors:** Caroline Rodrigues de Oliveira, Carolina Cabral Pereira da Costa, Cinthia Cristine Rosa Campos Medaber, Luiz Carlos Moraes França, Samira Silva Santos Soares, Norma Valéria Dantas de Oliveira Souza

**Affiliations:** I Universidade do Estado do Rio de Janeiro. Rio de Janeiro, Rio de Janeiro, Brazil; II Centro Universitário Anhanguera de Niterói. Niterói, Rio de Janeiro, Brazil; III Universidade Estadual de Santa Cruz. Ilhéus, Bahia, Brazil

**Keywords:** Nursing, Teaching, Emergency Remote Teaching, COVID-19, Stomatherapy., Enfermería, Enseñando, Enseñanza Remota de Emergencia, COVID-19, Estomaterapia.

## Abstract

**Objectives:**

to identify and analyze the perceptions of stomatherapy graduates regarding the advantages and disadvantages of in-person and emergency remote education during the COVID-19 pandemic.

**Methods:**

this qualitative, descriptive study was conducted at a public university in Rio de Janeiro with 28 graduates from the program. Data collection was carried out through semi-structured interviews, and the IRaMuTeQ^®^ software was used for data processing, specifically for lexical analysis.

**Results:**

the advantages of in-person education include the opportunity to clarify doubts, a close relationship between professors and students, and content enrichment through debate. The advantages of remote education include flexibility in location and schedule, reduced expenses, and less time spent in traffic congestion. Regarding the disadvantages of remote education, issues related to access to technology and a lack of skills in handling it emerged.

**Conclusions:**

the labor market, driven by neoliberal principles, strongly impacts the professional qualification process, presenting challenges and causing intense distress and psychophysical overload in both in-person and remote education.

## RESUMEN

**Objetivos:**

identificar y analizar la percepción de egresados de estomaterapia sobre las ventajas y desventajas de la enseñanza presencial y remota de emergencia durante la pandemia de COVID-19.

**Métodos:**

estudio cualitativo, descriptivo, desarrollado en una universidad pública de Río de Janeiro con 28 egresados del curso. La recopilación de datos se llevó a cabo mediante entrevistas semiestructuradas, y se utilizó el *software* IRaMuTeQ^®^ para el procesamiento de los datos, específicamente para el análisis léxico.

**Resultados:**

las ventajas de la enseñanza presencial incluyen la oportunidad de aclarar dudas, la relación cercana entre profesor y estudiante y el enriquecimiento del contenido a través del debate. Las ventajas de la enseñanza remota incluyen la flexibilidad en la ubicación y el horario, la reducción de gastos y la disminución de los embotellamientos de tráfico. Como desventajas de la enseñanza remota, surgieron problemas relacionados con el acceso a las tecnologías y la falta de habilidades en su manejo.

**Conclusiones:**

el mundo laboral, basado en preceptos neoliberales, impacta fuertemente el proceso de capacitación profesional, presentando factores que dificultan dicho proceso y generando intenso sufrimiento y sobrecarga psicofísica, tanto en la enseñanza presencial como en la remota.

## INTRODUCTION

The pandemic brought significant challenges to all aspects of societal life, with the educational field being particularly impacted. Academic calendars worldwide were readjusted, and in Brazil, in-person classes were immediately suspended, followed by the subsequent incorporation of Emergency Remote Education (ERE). In this context, substantial transformations were implemented, along with a profound and ongoing dialogue among faculty, students, and other collaborators involved in the teaching-learning process to overcome the challenges stemming from the necessity of using information and communication technologies (ICT)^(1)^.

In this scenario, ERE was adopted as a temporary and urgent strategy in response to the pandemic, aiming to ensure the continuity of ongoing academic semesters. This approach helped prevent gatherings and the spread of COVID-19 by utilizing ICT to promote safe teaching and learning for students, faculty, and other professionals involved in the educational process, allowing adaptation to the new national context^(2)^.

ICT comprises a set of technologies, devices, and systems used to process, store, transmit, and share information and data. These technologies facilitate the efficient collection, distribution, and sharing of information, encompassing elements such as software, hardware, networks, and mobile devices, including smartphones and tablets^(3)^. The integrated combination of these resources enables communication in various settings, including the teaching-learning context.

The abrupt changes in the educational process caused by the pandemic generated significant uncertainties among educational administrators, teachers, students, parents, and the scientific community-including nursing professionals-regarding the effectiveness of remote education compared to in-person education. It is worth noting that success in distance education depends on multiple factors, such as the student’s profile, motivation, access to the internet, technological resources, and the digital competence of instructors in this modality^(4)^.

It should be emphasized that, although remote education has presented obstacles and challenges to the teaching-learning process and the handling of ICT by both teachers and students, it has enabled the resumption or continuity of education. However, its use has also led to negative consequences, such as insecurity in using these technologies, the need for rapid adaptations in the home environment for teaching, the demand for psychocognitive skills due to the requirements of remote classes, and the intrusion of work and school into the domestic space^(5)^.

In nursing education, in-person instruction is essential for acquiring psychomotor and affective skills. In this regard, laboratories are available for both basic subjects (such as anatomy, biochemistry, physiology, microbiology, and parasitology) and specialized courses (such as nursing fundamentals, semiology, and semiotechnics). This context is highly favorable and relevant to the teaching-learning process in this profession^(6)^.

On the other hand, it should be noted that, in the case of stomatherapy qualification, remote education during the pandemic allowed for the continuation of the teaching-learning process and ensured the training of stomatherapists. Stomatherapy is a nursing specialty that trains professionals to care for individuals with wounds, stomas, and anal and urinary incontinence. There is a known unmet demand for this type of care, especially during the pandemic, when a significant increase in pressure injuries, colostomies, tracheostomies, ileostomies, and other conditions requiring specialized stomatherapist care was observed^(7)^.

Based on the literature review conducted in research databases on this subject, the relevance of this study is highlighted. It investigates the teaching-learning process in stomatherapy during an unprecedented period, such as a pandemic, and seeks to understand the challenges of in-person and ERE in this context, as well as how they can serve as a foundation for addressing future pandemics or atypical periods. Thus, presenting data for reflection on the complexity of teaching and learning in this profession and specialty in unusual times may point to new paradigms in education.

This study is an excerpt from the master’s dissertation of one of the authors, titled *In-Person and Remote Education During the COVID-19 Pandemic: Perceptions of Graduates from the Stomatherapy Program* ^(8)^.

## OBJECTIVES

To identify and analyze the perceptions of stomatherapy graduates regarding the advantages and disadvantages of in-person and ERE during the COVID-19 pandemic.

## METHODS

### Ethical Aspects

This research complied with the ethical and legal requirements for studies involving human subjects and was approved by the Research Ethics Committee of the respective university. Participant anonymity was ensured through the creation of a code generated after the consent process, consisting of the letter “P” for “participant,” followed by a cardinal number representing the order in which the interviews were conducted. Informed Consent Form (ICF) was obtained from all study participants, either in written or online format.

### Type of Study

This qualitative, descriptive, and exploratory study followed the Consolidated Criteria for Reporting Qualitative Research (COREQ) guidelines to structure the methodological approach^(9)^.

### Research Setting

The study was conducted at the nursing school of a public university in the state of Rio de Janeiro, where the investigated phenomenon was observed. In addition to undergraduate nursing education, the institution offers specialization courses, including a nursing program in stomatherapy.

Participants included 28 graduates from the stomatherapy nursing program at the respective university, originating from the firstand second-semester cohorts of 2019. The selection of these graduates was based on the fact that they had experienced both in-person education ERE during the most severe phase of the COVID-19 pandemic in 2020, a year marked by a rising number of cases and deaths caused by the disease and the absence of vaccines and/or effective treatments to combat it.

Excluded from the participant pool were nurses who had withdrawn from the course during the pandemic, as well as those who had not completed their qualification due to failure or dropout. These criteria were established to collect data from professionals who had fully experienced the teaching-learning process and had successfully completed their training.

### Data Collection and Organization

The data collection method used was the semi-structured individual interview, with an initial section gathering sociodemographic and professional information from the participants, followed by questions aimed at understanding the study’s subject matter. The data collection period occurred between August and September 2022, conducted either in person or remotely, depending on the participant’s preference and availability. Of the 28 interviews conducted, ten took place in person, while 18 were conducted remotely via telephone.

The participant characterization data included the following aspects: biological sex, marital status, age, race, cohort in which they attended the program, length of professional experience as a generalist, additional qualifications, practice as a stomatherapist, number and types of employment relationships, work settings as a nurse, and job title.

The interview script contained six core questions:

- Discuss your perception of in-person education.
- Discuss your perception of ERE in the stomatherapy specialization program, considering your learning experience.
- Describe the factors and/or situations that hindered your learning in the stomatherapy program in the remote modality.
- Describe the factors and/or situations that hindered your learning in the stomatherapy program in the in-person modality.
- Describe the factors and/or situations that facilitated your learning in the stomatherapy program in the remote modality.
- Describe the factors and/or situations that facilitated your learning in the stomatherapy program in the in-person modality.

### Data Analysis

The sociodemographic and professional characterization data of the participants were analyzed using descriptive statistics. Meanwhile, the information derived from the questions directly related to the study’s subject was processed using the *Interface de R pour les Analyses Multidimensionnelles de Textes et de Questionnaires* (IRaMuTeQ) software, which enables statistical analysis of texts, organizing vocabulary in a clear and comprehensible manner through lexical analysis^(10)^.

For this study, the descending hierarchical classification (DHC) method was employed, which separates or classifies text segments (TS) according to their respective lexicons, with the TS sets distributed based on lexical affinity^(11)^. Through this data processing, five classes emerged, among which the one that forms the basis of this study is titled “In-Person Education and Emergency Remote Education: Comparing Facilitators and Barriers”.

## RESULTS

### Participant characterization

Of the 28 participants (100%), 12 (42.85%) were from the first-semester cohort (2019.1), and 16 (57.14%) were from the second-semester cohort (2019.2). The age range varied from 27 to 55 years, with a predominance of participants aged between 30 and 40 years (N=10/35.71%). The majority were female (N=24/85.71%). Additionally, 12 (42.85%) were single.

Regarding self-reported race, 23 participants (82.14%) identified as Black or mixed race. In terms of professional characterization, considering the length of experience as a generalist nurse, 13 participants (46.42%) had graduated between two and five years prior. Six (21.42%) graduates worked as stomatherapy nurses, while 13 (46.42%) did not practice in the specialty, and nine (32.14%) were partially involved in the field, as they performed activities related to the care of individuals requiring stomatherapy but were not specifically employed in this role.

Regarding additional qualifications, the majority (N=13/46.42%) had completed another specialization besides stomatherapy. In terms of employment relationships, 17 (60.71%) had a single job, while 11 (39.28%) held more than one job. Furthermore, 21 (75%) were employed under the regulations of the Consolidation of Labor Laws.

### In-Person Education and ERE: comparing advantages and disadvantages

This category generated 176 out of a total of 799 lexicons, among which the most frequent were: Saturday (X^2^: 75.5), week (X^2^: 55.18), schedule (X^2^: 50.06), commitment (X^2^: 50.44), and shift (X^2^: 48.9), among others specified in Table 1.

**Table 1:** Presentation of the Most Frequent Lexicons in the Category

Order	Absolute frequency in class	Absolute frequency in corpus	%	x^2^	Type	Lexicon / form	*p* value
0	27	32	84.38	75.45	Nom	Saturday	<0.0001
1	18	20	90.0	55.18	Nom	Week	<0.0001
2	19	23	82.61	50.6	Nom	Schedule	<0.0001
3	14	14	100.0	50.44	Nom	Commitment	<0.0001
4	15	16	93.75	48.9	Nom	Shift	<0.0001
5	18	22	81.82	47.009	Nom	Commuting	<0.0001

*Source: IRaMuTeQ, 0.7 alpha 2, 2022.*

No standout variable was identified, suggesting that the advantages and challenges of both teaching modalities were discussed and compared by graduates from both cohorts, as well as by participants of both sexes.

The participant with the highest contribution was P25, from the 2019.1 cohort and female, with X^2^: 4.49 and 43.75% of their ST in the category, followed by P08, from the 2019.2 cohort and also female, with X^2^: 4.12 and 36.36% of their ST contributions.

This category highlighted both the challenges and facilitators of the teaching-learning process in in-person education and ERE, which were closely linked to the graduates’ roles in the workforce and the need to balance their professional activities with their qualification process. It also brought to light gender-related issues that impacted learning and teaching.

It was observed that, in order to obtain the specialist title, participants had to sacrifice their leisure time, rest, and family interactions. Additionally, the combination of work-related responsibilities with personal life activities (such as caring for family members) and the qualification process was identified as a complicating factor in both teaching modalities, especially for women, raising gender-related concerns.

*It means giving up every weekend, especially for those who work shifts like me, to obtain the stomatherapist title. No one trades a Saturday day shift for a Monday. So, I ended up working on Sundays, compromising both my Saturday and Sunday. This affected my rest and leisure time.* (P14)

*During the pandemic, I was afraid because I had elderly parents at home, and during the week, I had to commute to work since I continued working at the hospital, in addition to my commitment to finishing the course. I cared for patients and remained concerned about my elderly parents, and their care was added to the remote classes on Saturdays.* (P06)

Participants also perceived the financial cost of the course as a barrier to their qualification process, regardless of the teaching modality. This was particularly evident when considering their salaries, which were often deemed insufficient to cover their material needs and continue their professional qualification. However, participants stated that the financial burden was reduced in ERE since they did not have to bear transportation costs.

*It was difficult to afford the course; people end up dropping out midway because it gets too expensive. In addition to paying tuition, we often had to pay for a shift replacement to attend class every Saturday, and our salaries are terrible, both in the in-person and remote phases.* (P27)

When asked about the disadvantages of in-person education, one major challenge identified was the difficulty of balancing work and qualification. In some cases, participants had to choose between missing a class or attending work, especially when their work schedule was changed to weekends. This situation led to missed course content, negatively impacting the teaching-learning process.

*I missed important content because, since it was a full-day course, we lost a lot when we were on shift, especially when there were schedule changes. Sometimes, we would only have that content on that specific Saturday, so the challenge was balancing the work schedule with the qualification process.* (P08)

The adoption of ERE due to the COVID-19 pandemic facilitated specialization for participants by offering flexibility in how and where they attended classes. The highlighted ST below illustrates this situation.

*The convenience of not having to leave home, a day when I don’t have to wake up early, a day I can spend with my daughter. So, there are pros and cons to remote education, considering our daily routine. Thus, I cannot deny the convenience of not having to commute and being able to stay home with my family.* (P07)

Other positive aspects of remote education mentioned by participants included the absence of commuting, reduced transportation costs, and not having to face traffic congestion and long distances.

*Commuting to and arriving at the university is exhausting because some people live far away, use public transportation, and deal with traffic, which impacts in-person education and makes remote education more convenient.* (P20)

A key advantage of in-person education was the physical proximity to professors, the immediate ability to clarify doubts, and discussions between students and faculty about course content, which enhanced learning.

*Learning in an in-person setting was highly productive because, although it was tiring to work all week, I could clear up doubts at that moment-exactly at that moment-exchange ideas with other students, and learn much more. I preferred in-person classes.* (P08)

Regarding the disadvantages of ERE, challenges emerged, such as difficulties handling ICT, the quality of students’ internet connections, the inadequacy of physical space for attending remote classes at home, and the interference of family members during lessons, which required participants’ attention and involvement in household tasks.

*It was difficult because we spent two consecutive weeks without internet, and my data plan was very limited. So, I couldn’t attend classes and spent two entire Saturdays on the phone calling the service provider.* (P10)

*I was already doing this throughout the week in my professional life. So, when Saturday came, I wouldn’t turn on the camera-I would just listen to the lecture while doing other things around the house. Besides that, there was noise in the home environment and family requests that distracted me.* (P01)

## DISCUSSION

The COVID-19 pandemic abruptly and unprecedentedly imposed the need for radical modifications in social routines. In this context, ERE was an example of a shift in educational practices, as it provided an alternative to mitigate the negative impacts of the pandemic on the teaching-learning process and to meet the needs for training and qualification^(12)^.

This abrupt change revealed both advantages and challenges that allow for an analysis and comparison of teaching modalities, particularly when associated with the urgency of entering or remaining in the workforce and the personal demands of those undergoing this transitional process during their professional qualification.

From this perspective, the labor market is shaped by neoliberal principles, which strongly impact society as a whole, often generating intense distress. This economic model has brought changes for workers and labor organizations, leading to job insecurity, the loss of labor rights, inadequate wages, and uncertainty regarding future employment stability. Due to this configuration, multiple job-holding and competition among peers have become common, as professionals seek to demonstrate to employers that they are versatile, multifunctional, competent, and highly skilled^(13)^.

A study^(14)^ conducted in Brazil, also involving graduates of the stomatherapy program, found that nurses invest in specialization courses to advance their careers and secure employment and financial stability. They believe that obtaining additional qualifications will improve their job prospects in an increasingly demanding labor market. Furthermore, certain regions face an oversupply of healthcare professionals, driving individuals to develop strategies for establishing themselves in fields with greater opportunities, such as stomatherapy.

Moreover, professional qualification is a significant aspiration for many Brazilian nursing professionals, as it offers access to new and better employment opportunities, recognition, salary increases, professional appreciation, and other benefits^(15)^.

Within this framework, neoliberal ideals influence and impact the labor market, driving increased demand for specialization courses aimed at enhancing skills, competencies, and professional attitudes. While this enables professionals to compete for better job positions, it also results in overburdening, as they must balance training with work responsibilities, often managing more than one job^(14)^.

Given the specificities and complexities of the labor market, professionals increasingly need to pursue further qualifications. Such training also involves acquiring greater competence in performing job-related functions, fostering technical skills, initiative, autonomy, problem-solving abilities, and creativity. *Lato sensu* postgraduate programs^(14)^ are one of the pathways through which professionals can enhance their qualifications.

From this perspective, according to the characterization of participants captured in this study, 21 (75%) of the interviewees had completed another specialization in addition to stomatherapy. In order to meet the demands of the labor market and their aspirations for better working conditions and higher salaries-without neglecting family obligations or social and personal needs-participants reported that undertaking the course represented an additional physical and mental burden.

Another aspect highlighted, related to the labor market and the training process of nurses, was the phenomenon that occurred from the late 2000s onward, when there was an extraordinarily high and unregulated increase in nursing degree programs. This resulted in an excessive number of nurses, leading to fierce competition, deteriorating working conditions, and an increased need for further qualification to secure employment. Additionally, there has been a decline in the availability of job positions, unfavorable working conditions, low salaries, limited recognition, and professional devaluation^(16)^. This situation has had a direct impact on the continuity and completion of specialization courses, particularly due to reduced salaries, which lead to financial default or course abandonment.

In career development, it is essential to align individual needs with the demands of the labor market. The challenge lies in balancing personal aspirations and responsibilities with the requirements of the organizations in which professionals are employed, seeking equilibrium between personal growth and workplace expectations^(17)^.

The difficulties reported by participants in pursuing their professional qualification align with another study^(18)^ conducted in 2020, which revealed that the vast majority of nurses seek higher professional qualifications despite the significant challenges encountered in their academic journeys. The desire for specialization persists even in the face of obstacles that arise during training, such as a lack of time and the need to reconcile academic pursuits with family responsibilities, particularly for nurses working dual shifts.

Physically and psychologically, balancing work with a qualification process is demanding, especially considering the nature of nursing activities, which involve lifting heavy loads due to patient mobilization, standing for long hours in static postures, exposure to harmful microorganisms, and dealing with end-of-life situations, as seen during the COVID-19 pandemic^(19)^. Thus, the specific demands of this profession, combined with the academic requirements of qualification, make it particularly exhausting.

A study^(20)^ highlighted higher levels of distress and illness in the labor market among women, particularly during the COVID-19 pandemic, as they often had to juggle responsibilities related to motherhood, work, and academic studies. Consequently, while women seek professional qualification to maintain their positions in the workforce, they experience a significantly greater psychophysical burden compared to men.

It is understood that in-person education is crucial for the training and qualification of nurses, as it is a profession that requires psychomotor skills, involving technical procedures that need to be practiced and developed safely^(21)^. This form of education is essential in the field of nursing, primarily due to the need for human interaction, reinforcing the importance of a close relationship between faculty and students to facilitate the exchange of experiences and clarification of doubts, aiming not only for quality education but also for a socio-affective bond among those involved^(22,23)^.

One of the advantages of in-person education highlighted was the immediate resolution of doubts, which provides students with confidence in what they have learned. In this regard, it is asserted that dialogue and proximity allow students to express their questions and provide feedback on their learning. Additionally, these interactions enable instructors to identify students’ educational needs and deficits, allowing for a reassessment of the pedagogical strategies used^(23)^.

Interpersonal relationships are fundamental for students to establish connections and professional networks, facilitating job market entry and collaboration in addressing workplace challenges. From this perspective, interpersonal relationships fostered through in-person education play a crucial role in understanding, offering, and receiving support not only in the learning process but also in the professional environment^(24)^.

In addition to previous findings related to high work demands and family and social obligations, it is noteworthy that participants identified several advantages of remote education, particularly its flexibility in terms of how and where they could attend classes. Other positive aspects of remote education mentioned by participants included the absence of commuting, reduced expenses, and the elimination of the need to face traffic congestion and long distances.

Research on the benefits of remote education among nursing students supports this analysis, highlighting the extra time gained for studying and other activities due to the absence of commuting. Furthermore, studies cited flexibility in attending classes, reduced financial costs, increased autonomy in the teaching-learning process, and improvements in learning quality and critical-reflective thinking^(25,26)^.

Beyond the flexibility of schedules and geographic accessibility, a study^(1)^ asserts that ERE is dynamic and collaborative, fostering interpersonal relationships. Among the positive impacts of ERE, notable benefits include enhanced proficiency in handling ICT, as well as increased creativity and autonomy.

ICT was one of the cornerstones of the teaching-learning process during the COVID-19 pandemic. However, it also required training and technical skills from those involved in this process, making it a positive predictor of academic performance^(27)^.

This transition occurred abruptly, without sufficient time to implement the necessary infrastructure for the proper development of ERE. Additionally, both faculty and students were not adequately trained in the use and management of ICT^(28)^.

Regarding ICT, an important factor to consider is the limited proficiency, even among instructors, in managing these technologies. Many had little to no prior experience using such tools for conducting classes, applying them in some cases only as a supplementary activity. Moreover, it is essential to highlight that due to the economic hardship frequently experienced by nursing professionals, access to high-quality computers, reliable internet, and ergonomic furniture is not a reality for many. Therefore, the essential infrastructure for distance learning, such as that required for ERE, presents a challenge that cannot be overlooked in this context^(28)^.

Additionally, the pandemic exacerbated social and educational inequalities in Brazil, as ERE depends on digital platforms and ICT, which highlights technological disparities and unequal access to digital media across different regions of the country, closely tied to socioeconomic inequalities^(2)^. The concept of free access and digital inclusion remains underdeveloped due to structural issues such as a lack of access to computers, the internet, and an appropriate learning environment, resulting in the exclusion of the most vulnerable and the deepening of socio-educational disparities^(29)^.

### Study limitations

The main limitation of this study was its focus on investigating the perceptions of graduates from a single educational institution. Therefore, the results may not be generalizable, as this setting presents specific characteristics related to institutional culture, distinct influences from the labor market in the context of Rio de Janeiro, and unique aspects of the teaching-learning process. However, it is believed that this study can serve as a basis for rethinking teaching methodologies and identifying new educational paradigms.

### Contributions to the Field

Understanding graduates’ perceptions of this subject can contribute to improving stomatherapy education from the perspective of those directly involved. Additionally, this study may provide insights for course managers and coordinators, enabling deeper reflections and a better understanding of the diversity within the teaching-learning process.

## FINAL CONSIDERATIONS

The analysis of the perceptions of graduates from a stomatherapy specialization course revealed a preference for in-person education. Regarding ERE, it presents the following advantages: it provides opportunities for clarifying doubts about classroom content; allows for a closer relationship between professors and students; promotes the supplementation and enrichment of delivered content through student discussions; and fosters networking, given the interpersonal relationships established between students and faculty.

However, ERE also demonstrated some advantages, such as the ability to attend classes from home, reducing transportation expenses and the stress caused by traffic congestion, as well as the possibility of attending the course while simultaneously managing other tasks. The specific difficulties of ERE stem from a lack of proficiency in handling ICT, limited or low-quality internet access and other technologies, and distractions in the home environment, all of which compromise the learning process.

Both in-person education and ERE presented challenges, including psychophysical strain due to the need to balance coursework with professional and family responsibilities. Notably, female professionals face an additional burden as they juggle workplace duties with household tasks. Furthermore, the necessity for multiple employment contracts, driven by precarious working conditions in nursing and low wages, affects students’ ability to cover the costs of specialization programs along with personal and family expenses.

## Data Availability

Not applicable.

## References

[1] Capellari C, Herrmann LG, Kaiser DE, Mancia JR. (2022). Potentialities and difficulties in nursing education during the COVID-19 pandemic. Rev Gaúcha Enferm.

[2] Fernandes SF, Nunes RJ, Almeida Neta AG, Menezes HF, Melo KC, Freitas RJ (2021). [The use of emergency remote education during the covid-19 pandemic: experience of teachers in nursing higher education]. Saúde Redes.

[3] Gusso AK, Castro BC, Souza TN. (2021). Education and communication technologies in nursing teaching during the COVID-19 pandemic: integrative review. RSD.

[4] Maciel MA, Andreto LM, Ferreira TC, Mongiovi VG, Figueira MC, Silva SL (2020). The challenges of using active methodologies in remote teaching during the covid-19 pandemic in a higher nursing course: an experience report. Braz J Dev.

[5] Saraiva K, Traversini C, Lockmann K. (2020). [Education in times of COVID-19: remote teaching and teacher exhaustion]. Práxis Educativa.

[6] Varella TC, Carvalho EC, Andrade KB, Soares SS, Pereira SR, Farias SN (2021). [Nursing graduation in times of covid-19: reflections on technology-mediated education]. EaD Foco.

[7] Carvalho SO, Moura MC, Mesquita MK, Silva GA, Vasconcelos CD, Silva GR. (2021). Extension actions in stomatherapy: experience report during the pandemic. RDS.

[8] Oliveira CR. (2023). Ensino presencial e remoto em tempos de pandemia de covid-19: percepção de egressos do curso de estomaterapia.

[9] Souza VR, Marziale MH, Silva GT, Nascimento PL. (2021). Translation and validation into Brazilian Portuguese and assessment of the COREQ checklist. Acta Paul Enferm.

[10] Camargo BV, Justo AM. (2013). Iramuteq: a free software for analysis of textual data. Temas Psicol.

[11] Camargo BV, Justo AM. (2018). Tutorial para uso do software Iramuteq.

[12] Neves VN, Valdegil DA, Sabino RN. (2021). Ensino remoto emergencial durante a pandemia de Covid-19 no Brasil: estado da arte. Rev Pemo.

[13] Duarte JM, Simões AL. (2015). Meanings of work to nursing professionals at a teaching hospital. Rev Enferm UERJ.

[14] Costa CC, Soares SS, Vieira ML, Oliveira MD, Pedro RS, Chaves US (2021). Stomatherapists in the world of work: practicalities and difficulties for the professional practice. Esc Anna Nery.

[15] Silva MC, Machado MH. (2020). Health and Work System: challenges for the Nursing in Brazil. Cien Saude Col.

[16] Martins LK, Rodrigues RM, Souza RK, Conterno SF, Luz MS. (2019). [Expansion of graduation courses in nursing in Brazil between 2004 and 2017]. Enferm Foco.

[17] Santos AS, Oliveira CT, Dias AC. (2015). Characteristics of the relationship between college students and their peers: implications for adjustment to college. Psicol Teor Prat.

[18] Wojastyk LD, Paula MA, Prado MN. (2020). Stomatheraphy: influences and repercussions on the professional career. Estima.

[19] Santos TS, Bragagnollo GR, Tavares CM, Papaleo LK, Carvalho LW, Camargo RA. (2020). Qualificação profissional de enfermeiros da atenção primária à saúde e hospitalar: um estudo comparativo. Rev Cuid.

[20] Speroni GA, Artmann SK, Schultz CC, Rocha AS, Kolankiewicz AC, Stumm EM. (2021). Dor musculoesquelética em profissionais de saúde que atuam em um centro de triagem da Covid-19. SC.

[21] Costa R, Lino MM, Souza AI, Lorenzini E, Fernandes GC, Brehmer LC (2020). Nursing teaching in covid-19 times: how to reinvent it in this context?. Texto Contexto Enferm.

[22] Serra IV, Lima JM, Silva GT, Santos JX, Santana LS. (2022). Remote teaching during the covid-19 pandemic: a viewpoint according to Paulo Freire’s approach. Cogitare Enferm.

[23] Santos LR, Albuquerque CF, Veiga LL, Peixoto MA. (2021). [Emergency remote teaching in the perspective of metacognition: analysis of the students’ perception in a technical nursing course]. EaD Foco.

[24] Cesario JB, Ribeiro MR, Dias RB, Rotherbarth AA, Lima LP. (2016). Portfólio reflexivo como estratégia de avaliação formativa. Rev Baiana Enferm.

[25] Lopes EA. (2020). Vivências de sofrimento e adoecimento em ambiente de trabalho: uma análise do cotidiano profissional de enfermeiras e enfermeiros num contexto pandêmico em dois centros de referência no atendimento a pacientes de Covid-19. Cad Psicol Soc Trab.

[26] Irineu NB, Gallo AM, Furuya RK, Salvagioni DA, Roecker S, Araujo JP. (2021). Ensino-aprendizagem em tempos de pandemia por Covid-19: desafios e facilidades enfrentadas pelos estudantes. Braz J Health Rev.

[27] Lima MG, Silva KV, Santos RL, Martins AK, Bem MS, Leonel AC (2022). Tecnologias para o cuidado em saúde mental e enfermagem: revisão integrativa. RSD.

[28] Galvao MC, Ricarte IL, Darsie C, Foster AC, Ferreira JB, Carneiro M (2021). [Uses of information and communication technologies in nursinghigher education during the COVID-19 pandemic]. BRAJIS.

[29] Kantorski LP, Wünsch CG, Souza TT, Farias TA, Oliveira MM. (2022). Potentials and limits of remote emergency mental health teaching in the context of COVID-19. Rev Enferm UFSM.

