# Supplementary material for: Perceptions of stomatherapy graduates regarding in-person and remote education during COVID-19 pandemic
Source: Rev Bras Enferm. 2025 Aug 8;78(4):e20240215. doi: 10.1590/0034-7167-2024-0215 (PMC12334127; doi:10.1590/0034-7167-2024-0215)
Supplement: Translation

# Translation: Percepções de egressos de estomaterapia sobre o ensino presencial e remoto durante pandemia de COVID-19

**DOI:** 10.1590/0034-7167-2024-0215pt

**Authors:** Caroline Rodrigues de Oliveira, Carolina Cabral Pereira da Costa, Cinthia Cristine Rosa Campos Medaber, Luiz Carlos Moraes França, Samira Silva Santos Soares, Norma Valéria Dantas de Oliveira Souza

**Affiliations:** I Universidade do Estado do Rio de Janeiro. Rio de Janeiro, Rio de Janeiro, Brasil; II Centro Universitário Anhanguera de Niterói. Niterói, Rio de Janeiro, Brasil; III Universidade Estadual de Santa Cruz. Ilhéus, Bahia, Brasil

**Keywords:** Enfermagem, Ensino, Ensino Remoto Emergencial, COVID-19, Estomaterapia.

## Abstract

**Objetivos:**

identificar e analisar a percepção de egressos de estomaterapia sobre as vantagens e desvantagens do ensino presencial e remoto emergencial durante a pandemia de COVID-19.

**Métodos:**

estudo qualitativo, descritivo, desenvolvido em uma universidade pública do Rio de Janeiro, com 28 egressos do curso. A coleta de dados foi realizada por meio de entrevista semiestruturada, e utilizou-se o *software* IRaMuTeQ^®^ para o tratamento dos dados, especificamente a análise lexical.

**Resultados:**

as vantagens do ensino presencial incluem a oportunidade para esclarecer dúvidas, a relação estreita entre professor e estudante e o enriquecimento do conteúdo pelo debate. As vantagens do ensino remoto incluem a flexibilidade de local e horário, a diminuição de gastos e a redução de congestionamentos no trânsito. Como desvantagens do ensino remoto, emergiram questões relacionadas ao acesso às tecnologias e à falta de habilidade no seu manuseio.

**Conclusões:**

o mundo do trabalho, pautado por preceitos neoliberais, impacta fortemente o processo de qualificação profissional, apresentando fatores dificultadores e gerando sofrimento intenso e sobrecarga psicofísica, tanto no ensino presencial quanto no remoto.

## INTRODUÇÃO

O contexto pandêmico trouxe mudanças desafiadoras em todas as vertentes da vida em sociedade, destacando-se o campo pedagógico. Os calendários acadêmicos, globalmente, foram readequados e, no caso do Brasil, observaram-se a imediata paralisação das aulas presenciais e a posterior incorporação do Ensino Remoto Emergencial (ERE). Nessa perspectiva, impuseram-se transformações contundentes e, ao mesmo tempo, um diálogo profundo e permanente entre docentes, discentes e demais colaboradores envolvidos no processo de ensino-aprendizagem para superar os desafios decorrentes da necessidade de uso das tecnologias da informação e comunicação (TIC)^(1)^.

Nesse contexto, o ERE foi a estratégia adotada de forma temporária e urgente em resposta ao cenário pandêmico, com o objetivo de garantir a continuidade dos semestres letivos em andamento. Dessa forma, evitaram-se aglomerações e a disseminação da COVID-19, utilizando-se as TIC para promover, de modo seguro, o ensino e a aprendizagem para discentes, docentes e demais profissionais envolvidos no processo educacional, permitindo a adaptação ao novo contexto nacional^(2)^.

As TIC constituem um conjunto de tecnologias, dispositivos e sistemas usados para processar, armazenar, transmitir e compartilhar informações e dados, englobando tecnologias que coletam, distribuem e compartilham informações de maneira eficaz, abrangendo elementos como *softwares*, hardwares, redes e dispositivos móveis, como celulares e tablets^(3)^. A combinação integrada desses recursos viabiliza a comunicação em diversos cenários, inclusive no contexto do ensino-aprendizagem.

As mudanças abruptas no processo educacional geradas pela pandemia originaram muitas incertezas para gestores pedagógicos, professores, estudantes, pais e a comunidade científica - incluindo a de enfermagem - sobre a eficácia do ensino não presencial em relação ao ensino presencial. Ressalta-se que o êxito no ensino a distância depende de muitos fatores, como o perfil do estudante, sua motivação, seu acesso à internet e seus recursos tecnológicos, além da formação e da competência digital dos professores para a docência nessa modalidade de ensino^(4)^.

Salienta-se que o ensino remoto, por mais que tenha apresentado obstáculos e desafios para o processo de ensino-aprendizagem e para o manuseio das TIC, tanto para docentes quanto para discentes, possibilitou a retomada ou a continuidade do ensino. Entretanto, seu uso fez emergir repercussões nem sempre positivas, como o sentimento de insegurança na utilização dessas tecnologias, a necessidade de adaptações rápidas do ambiente doméstico para a ministração de aulas, a mobilização de habilidades psicocognitivas diante da exigência de aulas remotas e a invasão da casa pelo trabalho e pela escola^(5)^.

No ensino de enfermagem, a modalidade presencial é indispensável para a aquisição de habilidades psicomotoras e afetivas. Nessa perspectiva, há, inclusive, a disponibilidade de laboratórios para disciplinas básicas (como anatomia, bioquímica, fisiologia, microbiologia e parasitologia) e específicas (como fundamentos de enfermagem, semiologia e semiotécnica). Desse modo, trata-se de um contexto altamente favorável e pertinente ao processo de ensino-aprendizagem dessa profissão^(6)^.

Por outro lado, ressalta-se que, no caso da qualificação em estomaterapia, o ensino remoto no contexto da pandemia possibilitou a continuidade do processo de ensino-aprendizagem e garantiu a formação de estomaterapeutas. A estomaterapia é uma especialidade da enfermagem que capacita os profissionais para cuidar de pessoas com feridas, estomas e incontinências anais e urinárias. Sabe-se que há uma demanda reprimida da população que carece dessa assistência, sobretudo em tempos de pandemia, quando se registrou um aumento significativo de lesões por pressão, colostomias, traqueostomias, ileostomias, entre outros eventos que exigiam a atuação do estomaterapeuta para promover um cuidado especializado^(7)^.

Considerando o levantamento literário nas bases de dados sobre a temática, destaca-se a relevância do estudo, que investiga o processo de ensino-aprendizagem na estomaterapia em tempos insólitos, como uma pandemia, e busca entender os desafios das modalidades de ensino presencial e remoto emergencial durante esse contexto, bem como a forma como podem servir de subsídio para o enfrentamento de novas pandemias ou tempos atípicos. Dessa forma, apresentar dados para reflexão sobre a complexidade que envolve o ensinar e o aprender na profissão e na especialidade, em momentos incomuns, pode apontar para novos paradigmas no campo do ensino.

Este estudo é um recorte da dissertação de mestrado de um dos autores, intitulada “*Ensino presencial e remoto em tempos de pandemia de COVID-19: percepção dos egressos do curso de estomaterapia”* ^(8)^.

## OBJETIVOS

Identificar e analisar a percepção de egressos de estomaterapia sobre as vantagens e desvantagens do ensino presencial e remoto emergencial durante a pandemia de COVID-19.

## MÉTODOS

### Aspectos éticos

Esta pesquisa respeitou as exigências éticas e legais para um estudo com seres humanos e foi aprovada pelo Comitê de Ética em Pesquisa da referida universidade. Também foi garantido o anonimato dos participantes por meio da criação de um código gerado após o processo de consentimento, caracterizado pela adição da letra “P”, de “participante”, seguida de um número cardinal que retrata a ordem na qual as entrevistas foram coletadas. O Consentimento Livre e Esclarecido foi obtido de todos os indivíduos no estudo por meio escrito ou online.

### Tipo de estudo

Este estudo, de natureza qualitativa, descritiva e exploratória, seguiu os critérios do *Consolidated Criteria for Reporting Qualitative Research* (COREQ) para nortear o desenvolvimento da metodologia^(9)^.

### Campo de pesquisa

O campo de pesquisa foi a faculdade de enfermagem de uma universidade pública no estado do Rio de Janeiro, local onde o fenômeno investigado foi observado. A referida instituição, além da graduação em enfermagem, oferece cursos de especialização, entre eles o curso de enfermagem em estomaterapia.

Os participantes foram 28 egressos do curso de enfermagem em estomaterapia da referida universidade, oriundos das turmas do primeiro e do segundo semestres de 2019. A escolha desses egressos fundamentou-se no fato de que eles vivenciaram o ensino presencial e o ERE durante a fase mais severa da pandemia de COVID-19 em 2020, ano marcado por um número crescente de casos e óbitos provocados pela doença e pela inexistência de vacinas e/ou tratamentos eficazes para combatê-la.

Foram excluídos do escopo de participantes enfermeiros que trancaram o curso durante a pandemia, bem como aqueles que não concluíram a qualificação por motivo de reprovação ou abandono. Esses critérios basearam-se no propósito de coletar dados com profissionais que vivenciaram integralmente o processo de ensino-aprendizagem e que tiveram êxito nesse percurso.

### Coleta e organização dos dados

A técnica de coleta de dados foi a entrevista individual semiestruturada, cuja parte inicial capturou dados sociodemográficos e profissionais dos participantes, seguida de questionamentos voltados à apreensão do objeto de estudo. O período de coleta ocorreu entre agosto e setembro de 2022, de forma presencial e/ou remota, de acordo com a preferência e disponibilidade do participante. Dessa forma, de um total de 28 entrevistas realizadas, dez ocorreram presencialmente e 18 remotamente, por ligação telefônica.

Os dados de caracterização dos participantes envolveram os seguintes aspectos: sexo biológico, estado civil, idade, cor, turma em que frequentou o curso, tempo de formação profissional como generalista, formações complementares, atuação como estomaterapeuta, quantidade e tipos de vínculos empregatícios, locais de atuação como enfermeiro e cargo ocupado.

O roteiro de entrevista continha seis questionamentos básicos:

- Fale sobre sua percepção acerca do ensino presencial;
- Fale sobre sua percepção acerca do ERE no curso de especialização em estomaterapia, considerando seu aprendizado;
- Discorra sobre fatores e/ou situações que dificultaram seu aprendizado no curso de estomaterapia na modalidade remota;
- Discorra sobre fatores e/ou situações que dificultaram seu aprendizado no curso de estomaterapia na modalidade presencial;
- Discorra sobre fatores e/ou situações que facilitaram seu aprendizado no curso de estomaterapia na modalidade remota;
- Discorra sobre fatores e/ou situações que facilitaram seu aprendizado no curso de estomaterapia na modalidade presencial.

### Análise dos dados

Os dados referentes à caracterização sociodemográfica e profissional dos participantes foram tratados por meio de estatística descritiva. Já as informações oriundas dos questionamentos ligados diretamente ao objeto de estudo foram processadas pelo *software Interface de R pour les Analyses Multidimensionnelles de Textes et de Questionnaires* (IRaMuTeQ), que permite realizar análises estatísticas sobre textos, organizando o vocabulário de forma compreensível e clara, por meio da análise lexical^(10)^.

Nesta pesquisa, utilizou-se a *classificação hierárquica descendente* (CHD), que separa ou classifica segmentos de texto (ST) de acordo com seus respectivos léxicos, sendo que o conjunto desses ST é repartido conforme a afinidade lexical^(11)^. Por meio desse processamento dos dados, originaram-se cinco classes, dentre as quais se destaca a que fundamenta este estudo, denominada “*Ensino presencial e ensino remoto emergencial: comparando facilidades e dificuldades”*.

## RESULTADOS

### Caracterização dos participantes

Dos 28 participantes (100%), 12 (42,85%) eram oriundos das turmas do primeiro semestre (2019.1) e 16 (57,14%) da turma do segundo semestre (2019.2). A faixa etária variou de 27 a 55 anos, com predominância entre 30 e 40 anos (N=10/ 35,71%). A maioria era do sexo feminino (N=24/ 85,71%). Além disso, 12 (42,85%) eram solteiros.

Em relação à autodeclaração de cor, 23 participantes (82,14%) se identificaram como pretos ou pardos.

No que se refere à caracterização profissional, considerando o tempo de formação como enfermeiro generalista, 13 (46,42%) participantes haviam se graduado entre dois e cinco anos. Seis (21,42%) egressos atuavam como enfermeiros estomaterapeutas, enquanto 13 (46,42%) não exerciam a especialidade e nove (32,14%) atuavam parcialmente na área, pois realizavam atividades relacionadas aos cuidados com pessoas em situação de estomaterapia, mas sem serem contratados especificamente para a função.

Quanto à formação complementar, a maioria (N=13/ 46,42%) realizou outra especialização além da estomaterapia. No que tange ao vínculo laboral, 17 (60,71%) possuíam um vínculo empregatício, enquanto 11 (39,28%) tinham mais de um vínculo. Ademais, 21 (75%) estavam empregados sob o regime da Consolidação das Leis do Trabalho (CLT).

### Ensino presencial e ERE: comparando vantagens e desvantagens

Esta classe gerou 176 de um total de 799 léxicos, dentre os quais os principais foram: *sábado* (X^2^: 75,5), *semana* (X^2^: 55,18), *horário* (X^2^: 50,06), *compromisso* (X^2^: 50,44) e *plantão* (X^2^: 48,9), entre outros especificados na Tabela 1.

**Tabela 1:** Apresentação dos léxicos mais frequentes da classe

Ordem	Frequência absoluta na classe	Frequência absoluta no *corpus*	%	X2	Tipo	Léxico / Forma	Valor de *p*
0	27	32	84,38	75,45	Nom	sábado	<0,0001
1	18	20	90,0	55,18	Nom	semana	<0,0001
2	19	23	82,61	50,6	Nom	horário	<0,0001
3	14	14	100,0	50,44	Nom	compromisso	<0,0001
4	15	16	93,75	48,9	Nom	plantão	<0,0001
5	18	22	81,82	47,009	Nom	deslocamento	<0,0001

*Fonte: IRaMuTeQ, 0.7 alpha 2, 2022.*

Não houve variável de destaque, o que permite inferir que as facilidades e dificuldades das duas modalidades de ensino foram abordadas e comparadas pelos egressos de ambas as turmas, bem como por participantes de ambos os sexos.

O participante com maior contribuição foi P25, da turma 2019.1 e do sexo feminino, com X^2^: 4,49 e 43,75% de seus ST na classe, seguido por P08, da turma 2019.2 e também do sexo feminino, com X^2^: 4,12 e 36,36% de contribuições dos ST.

Esta classe evidenciou situações dificultadoras e facilitadoras do ensino-aprendizagem, na modalidade presencial e no ERE, fortemente envolvidas com a atuação dos egressos no mundo do trabalho e a necessidade de associar suas atividades laborais ao processo de qualificação. Também fez emergir questões relacionadas ao gênero feminino, que impactavam o aprendizado e o ensino.

Apreendeu-se que, para alcançar o título de especialista, os participantes sacrificavam os dias de lazer, descanso e convívio com a família. Além disso, a conjugação de questões laborais com atividades da vida pessoal (como o cuidado aos familiares) e com o processo de qualificação foi apontada, em ambas as modalidades de ensino, como um fator dificultador, sobretudo para as mulheres, remetendo a questões de gênero.

*É comprometer todos os finais de semana, ainda mais para quem é plantonista, como eu, para obter o título de estomaterapeuta. Ninguém troca um plantão de sábado de dia por uma segunda. Então, eu acabava trocando pelo domingo e comprometia o sábado e o domingo. Isso compromete meu descanso e lazer.* (P14)

*Durante a pandemia, eu tinha medo porque tinha meus pais idosos em casa, e, durante a semana, me deslocava para o trabalho, pois não parei de trabalhar no hospital, além do compromisso de finalizar o curso. Cuidava dos doentes e ainda ficava preocupada com meus pais idosos, e o cuidado com eles se somava às aulas remotas aos sábados*. (P06)

Os participantes também entenderam que o custo financeiro do curso foi um dificultador nesse processo de qualificação, independentemente da modalidade de ensino, especialmente ao se levar em conta seus salários, muitas vezes considerados insuficientes para pagar suas necessidades materiais e dar continuidade ao processo de qualificação profissional. No entanto, os participantes asseveraram que o custo financeiro foi minimizado no ERE, uma vez que não precisavam arcar com despesas de transporte.

*Era um dificultador pagar o curso; a gente acaba desistindo no meio do percurso porque fica caro. Além de pagar a mensalidade, muitas vezes tínhamos que pagar o plantão para comparecer todos os sábados, e nossos salários são péssimos, tanto na fase presencial quanto na remota.* (P27)

Quando perguntados sobre as desvantagens do ensino presencial, um dificultador apresentado foi a necessidade de conciliar o trabalho com a qualificação. Nesse sentido, algumas vezes os participantes tinham que escolher entre faltar ao curso e comparecer ao trabalho, principalmente quando a escala de trabalho era alterada para o final de semana. Tal situação resultava em perdas do conteúdo desenvolvido no curso, causando prejuízos para o processo de ensino-aprendizagem.

*Perdia um conteúdo importante, porque, como é o dia inteiro, a gente perde muita coisa quando está de plantão, principalmente considerando mudanças na escala. Às vezes, a gente só teria aquele conteúdo naquele sábado, então essa era a dificuldade: conciliar a escala de trabalho com a qualificação.* (P08)

A adoção do ERE devido à pandemia de COVID-19 gerou facilidades para os participantes cursarem a especialização, devido à flexibilização em relação à forma e ao local de assistir às aulas. O ST destacado a seguir evidencia essa situação.

*A facilidade de não ter que sair de casa, um dia em que já posso não acordar cedo, um dia que tenho para ficar com minha filha. Então, há prós e contras nesse ensino remoto, considerando nossa rotina de vida. Assim, não posso negar essa facilidade de não precisar me deslocar e de poder permanecer em casa com a família.* (P07)

Outros pontos positivos referidos, advindos do ensino remoto, foram a ausência de deslocamento, a redução dos gastos com transporte e o fato de não precisar enfrentar engarrafamentos e longas distâncias.

*Deslocar-se e chegar à faculdade é desgastante, porque algumas pessoas moram longe, utilizam transporte público, enfrentam trânsito, o que se torna um impacto no ensino presencial e um facilitador no ensino remoto*. (P20)

Um aspecto facilitador do ensino presencial foi a proximidade física dos professores, a possibilidade de esclarecer dúvidas imediatamente e as discussões entre discentes e docentes sobre os conteúdos do curso, o que favorece o aprendizado.

*O aprendizado em relação ao ensino presencial era muito produtivo porque, apesar de cansativo por trabalhar a semana toda, eu conseguia tirar as dúvidas ali naquele momento, exatamente naquele momento, trocar ideias com os outros alunos e aprender muito mais. Eu preferia o presencial.* (P08)

No que se refere às desvantagens do ERE, emergiram dificuldades como o manuseio das TIC, a qualidade da internet dos discentes, a inadequação do espaço físico para assistir às aulas remotas em seus domicílios e a interferência dos membros da família durante as aulas, que demandavam tarefas e atenção dos egressos.

*Foi difícil, porque ficamos duas semanas seguidas sem internet, e o meu pacote de dados era bem limitado. Então, eu não conseguia assistir às aulas e passei dois sábados inteiros no telefone, ligando para a operadora.* (P10)

*Porque eu já fazia isso durante a semana na minha vida profissional. Então, quando chegava o sábado, eu não abria a câmera, apenas ouvia a aula enquanto fazia outras coisas dentro de casa. Além disso, havia ruídos no ambiente doméstico e solicitações dos familiares que tiravam minha atenção.* (P01)

## DISCUSSÃO

A pandemia da COVID-19 impôs, de maneira abrupta e nunca antes vivenciada, a necessidade de realizar modificações radicais no cotidiano social. Nessa tessitura, o ERE foi um exemplo de mudança no cotidiano educacional, pois representou uma alternativa para mitigar os impactos negativos da pandemia no processo de ensino-aprendizagem e para atender às necessidades de formação e qualificação^(12)^.

Essa mudança abrupta descortinou facilidades e dificuldades que permitem analisar e comparar as modalidades de ensino, especialmente quando associadas à premência de inserção ou manutenção no mundo do trabalho e às demandas pessoais dos que vivenciaram esse processo de transição durante a qualificação profissional.

Por esse ângulo, entende-se que o mundo do trabalho é pautado por preceitos neoliberais, que impactam fortemente a sociedade como um todo, muitas vezes gerando sofrimento intenso. Esse modelo econômico trouxe mudanças para o trabalhador e para as organizações laborais e tem provocado precarização do trabalho, perda de direitos trabalhistas, salários indignos e insegurança em relação ao futuro, pois não há certeza da estabilidade no emprego. Em razão dessa configuração, verificam-se o multiemprego e a competitividade entre os pares, no sentido de evidenciar, para os empregadores, que se é polivalente, multifuncional, competente e habilidoso^(13)^.

Em um estudo^(14)^ conduzido no Brasil, também com egressos do curso de estomaterapia, constatou-se que os enfermeiros, para desenvolver a carreira e garantir emprego e estabilidade financeira, têm investido em especializações. Com isso, acreditam que obterão melhores colocações no mercado de trabalho, que atualmente apresenta um elevado nível de exigência profissional. Além disso, há regiões que se encontram saturadas de mão de obra, o que impulsiona o profissional a adotar estratégias para empreender em áreas mais propícias a esse fim, como a estomaterapia.

Ademais, a qualificação profissional é o anseio de muitos profissionais de enfermagem brasileiros, pois possibilita novas e melhores oportunidades de emprego, reconhecimento, aumento salarial, valorização profissional, entre outros benefícios^(15)^.

Nessa tessitura, o ideário neoliberal influencia e impacta o mundo do trabalho, sendo responsável pelo aumento da procura por especializações, com vistas ao aprimoramento de habilidades, competências e atitudes. Desse modo, possibilita ao profissional competir por melhores postos de trabalho; porém, acaba por sobrecarregá-lo, uma vez que precisa conciliar capacitação e trabalho, às vezes até com mais de um vínculo laboral^(14)^.

Diante das particularidades e complexidades do mundo do trabalho, o profissional precisa se qualificar cada vez mais. Tal qualificação perpassa também pela aquisição de maior competência no exercício das funções laborais, possibilitando o desenvolvimento da capacidade técnica, iniciativa, autonomia, resolução de problemas e criatividade. A pós-graduação lato sensu^(14)^ é um dos meios pelos quais o profissional pode se qualificar.

Nessa perspectiva, segundo a caracterização dos participantes capturada neste estudo, 21 (75%) dos entrevistados concluíram outra especialização além da estomaterapia. Para dar conta das exigências do mundo laboral e das aspirações por melhores condições de trabalho e salários mais atrativos, sem negligenciar as exigências familiares nem as necessidades sociais e pessoais, os participantes referiram que a realização do curso representou mais uma sobrecarga física e mental.

Outro aspecto pontuado, que envolve o mundo do trabalho e o processo de formação dos enfermeiros, foi o fenômeno ocorrido a partir do final dos anos 2000, quando houve um crescimento elevadíssimo e desordenado de cursos de graduação em enfermagem. Isso resultou em uma exorbitante disponibilidade de enfermeiros, gerando competitividade acirrada, precarização das condições de trabalho e necessidade de qualificação para a garantia do emprego. Ademais, observam-se a diminuição da oferta de postos de trabalho, condições laborais indignas, baixos salários, pouco reconhecimento e desvalorização profissional^(16)^. Essa situação tem impacto direto na continuidade e na conclusão do curso de especialização, especialmente devido aos salários reduzidos, o que resulta em inadimplência ou abandono do curso.

No desenvolvimento da carreira, é essencial alinhar as necessidades individuais com as exigências do mundo do trabalho. O desafio consiste em conciliar as demandas e aspirações pessoais com as exigências das organizações laborais nas quais se está inserido, buscando um equilíbrio entre o desenvolvimento pessoal e as expectativas do local de trabalho^(17)^.

As dificuldades relatadas pelos participantes para investir em sua qualificação profissional estão em consonância com outro estudo^(18)^, realizado em 2020, que evidenciou que a grande maioria dos enfermeiros busca maior qualificação profissional, apesar das repercussões na vida pessoal e profissional constituírem um verdadeiro desafio em suas trajetórias acadêmicas. O interesse pela especialização existe mesmo diante dos obstáculos que surgem no momento de se capacitar, como a falta de tempo e a necessidade de conciliação com as demandas familiares, especialmente no caso dos enfermeiros com dupla jornada de trabalho.

Física e psiquicamente, é custoso articular o trabalho com um processo de qualificação, sobretudo considerando as características das atividades laborais da enfermagem, que envolvem carregar peso devido à mobilização do paciente, permanecer por várias horas em pé e em posturas estáticas, ter contato com microrganismos perigosos à saúde e lidar com a finitude da vida, como ocorreu no caso da COVID-19^(19)^. Desse modo, a especificidade desse trabalho e as demandas acadêmicas da qualificação caracterizam-se, de fato, como desgastantes.

Um estudo^(20)^ evidenciou maior sofrimento e adoecimento no mundo do trabalho entre as mulheres, principalmente durante a pandemia de COVID-19, pois, muitas vezes, acumulavam as tarefas da maternidade, do trabalho e dos estudos. Assim, na tentativa de manter-se no mundo do trabalho, as mulheres buscam a qualificação profissional, porém vivenciam um custo psicofísico significativamente maior do que os homens.

Entende-se que o ensino presencial é pertinente à formação e qualificação do enfermeiro, pois trata-se de uma profissão que demanda habilidade psicomotora, envolvendo procedimentos técnicos que precisam ser treinados e desenvolvidos de forma segura^(21)^. Esse ensino apresenta-se como imprescindível na área da enfermagem, principalmente pela necessidade de contato humano, reafirmando a importância da relação próxima entre docente e estudante para que haja troca de experiências e esclarecimento de dúvidas, buscando não somente um bom ensino, mas também uma relação socioafetiva entre os envolvidos^(22,23)^.

Uma das vantagens do ensino presencial apontada foi a imediata retirada de dúvidas, o que confere ao estudante segurança em relação ao que aprendeu. Sobre esse ponto, assevera-se que o diálogo e a proximidade permitem que o discente exponha suas dúvidas e forneça feedback sobre seu aprendizado, além de possibilitar que o docente identifique as necessidades e os déficits do ensino dos estudantes, reavaliando as estratégias pedagógicas utilizadas^(23)^.

As relações interpessoais são fundamentais para que os acadêmicos criem vínculos e redes de contato (*network*), favorecendo a inserção no mercado de trabalho e a cooperação na busca de soluções para problemas do campo laboral. Sob essa ótica, as relações interpessoais oriundas do ensino presencial desempenham um papel imprescindível para a compreensão, a oferta e o recebimento de ajuda não apenas no aprendizado, mas também no mundo do trabalho^(24)^.

Associado às constatações anteriores de elevada demanda de trabalho e exigências familiares e sociais, merece destaque o fato de os participantes terem elencado pontos favoráveis ao ensino remoto devido à flexibilização em relação à maneira e ao local de assistir às aulas. Outros pontos positivos advindos do ensino remoto foram referidos pelos participantes, tais como a ausência de deslocamento, a redução dos gastos e a eliminação da necessidade de enfrentar engarrafamentos e longas distâncias.

Pesquisas sobre as facilidades do ensino remoto com discentes de enfermagem ratificam essa análise, mencionando o ganho de tempo extra para os estudos e outras atividades devido à ausência de deslocamento. Ademais, citaram a flexibilidade na forma de assistir às aulas, a redução de custos financeiros, o desenvolvimento da autonomia no processo de ensino-aprendizagem e a melhora na qualidade do aprendizado e do pensamento crítico-reflexivo^(25,26)^.

Para além da flexibilidade de horários e espaços geográficos, um estudo^(1)^ assevera que o ERE é dinâmico e colaborativo, permitindo relações interpessoais. Entre os impactos positivos do ERE, destacam-se a ampliação da habilidade no manejo das TIC, bem como o aumento da criatividade e da autonomia.

As TIC foram um dos alicerces da continuidade do processo de ensino-aprendizagem em tempos de pandemia da COVID-19. Em contrapartida, demandaram treinamento e habilidades dos atores envolvidos nesse processo para seu uso e manuseio, caracterizando-se, inclusive, como fator preditor positivo para a performance acadêmica^(27)^.

Essa migração ocorreu abruptamente, sem tempo suficiente para a instalação de infraestrutura condizente com o bom desenvolvimento do ERE. Ademais, os corpos docente e discente não foram adequadamente capacitados para o uso e a manipulação das TIC^(28)^.

Em relação às TIC, um aspecto importante que precisa ser ponderado é a pouca habilidade, inclusive dos docentes, no manuseio dessas tecnologias, pois tiveram pouco ou nenhum contato com tais ferramentas para a ministração das aulas, utilizando-as, em alguns casos, apenas como atividade complementar. Além disso, é fundamental salientar que, devido à hipossuficiência econômica frequentemente presente entre os profissionais de enfermagem, o acesso a bons computadores, internet potente e mobiliário ergonômico não é uma realidade para muitos. Portanto, a infraestrutura essencial para o aprendizado à distância, como ocorre no ERE, é uma dificuldade que não pode ser negligenciada nesse contexto^(28)^.

Complementando, a pandemia agravou as desigualdades sociais e educacionais no Brasil, pois, no caso do ERE, depende-se do uso de plataformas digitais e das TIC, o que evidencia a disparidade tecnológica e de acesso às mídias sociais em diferentes regiões do país, relacionada às desigualdades socioeconômicas^(2)^. A ideia de acesso livre e de inclusão digital ainda é incipiente, em decorrência de questões estruturais, como a falta de acesso a computadores, à internet e a um local apropriado para o aprendizado, resultando na exclusão dos mais vulneráveis e na acentuação das desigualdades socioeducativas^(29)^.

### Limitações do estudo

A limitação deste estudo foi investigar as percepções dos egressos em um único local de ensino. Logo, os resultados podem não ser generalizáveis, uma vez que esse cenário apresenta especificidades que envolvem a cultura institucional, influências diferenciadas do mundo do trabalho no contexto fluminense e características próprias do processo de ensino-aprendizagem. No entanto, acredita-se que este estudo pode servir como subsídio para repensar metodologias de ensino e apontar novos paradigmas educacionais.

### Contribuições para a área

Compreender a percepção dos egressos acerca dessa temática pode contribuir para o aprimoramento do ensino da estomaterapia a partir do ponto de vista desses sujeitos. Considera-se também que este estudo poderá fornecer subsídios para gestores e coordenadores dos cursos, permitindo reflexões e aprofundamento sobre a diversidade do processo de ensino-aprendizagem.

## CONSIDERAÇÕES FINAIS

A análise da percepção dos egressos de um curso de especialização em estomaterapia evidenciou que há preferência pelo ensino presencial e que, em relação ao ERE, este apresenta as seguintes vantagens: oportuniza o esclarecimento de dúvidas sobre o conteúdo desenvolvido em sala de aula; permite uma relação mais estreita entre professor e estudante; favorece a complementação e o enriquecimento do conteúdo ministrado por meio do debate entre os discentes; e estimula o *networking*, dada a relação interpessoal formada entre discentes e docentes.

No entanto, o ERE também apresentou algumas vantagens, como a possibilidade de assistir às aulas no domicílio, minimizando gastos com transporte e o desgaste causado pelo congestionamento no trânsito, além da possibilidade de frequentar o curso e desenvolver outras tarefas simultaneamente. As dificuldades específicas do ERE decorrem do déficit no manuseio das TIC, da falta ou baixa qualidade de acesso à internet e às demais tecnologias, além das distrações do ambiente doméstico, que comprometem o processo de aprendizagem.

Evidenciaram-se, tanto no ensino presencial quanto no ERE, dificuldades como o desgaste psicofísico inerente à conciliação do curso com as atividades laborais e as demandas familiares, com destaque para a sobrecarga das profissionais do sexo feminino, que acumulam funções no mundo do trabalho com as tarefas domésticas. Além disso, a necessidade de múltiplos vínculos empregatícios, decorrente da precarização do trabalho em enfermagem e dos baixos salários, impacta a capacidade do estudante de arcar com os custos da especialização e com os gastos pessoais e familiares.

## Data Availability

Não se aplica.
